# Medical and Physical Intelligence

**Published:** 1807-02

**Authors:** 


### MEDICAL AND PHYSICAL INTELLIGENCE.

The Copleyan Medal has been adjudged to T. A. KNIGHT, Esq. for his numerous discoveries in vegetable physiology. Sir Joseph Banks, upon presenting Mr. Knight with the reward of his labours and high merit, pronounced a most able discourse on the pursuits of this gentleman. He noticed his researches and observations on the albuminous juice of plants, in its *ascent* elaborating the buds and leaves, and in its *descent* forming wood: and of his discovery of the natural decay of apple-trees, and of the grafts, which decline and become unproductive at the same time with the parent stock. The learned President referred next to the experiments, which went to prove that all vegetables radiate by gravitation only, and not by any instinctive energy: that new and superior species of apples may be produced from seed: and that impregnating the pollen was found to be an advantageous substitute for grafting. He then alluded to the new and very valuable species of pears produced by Mr. Knight, and to a new species of vines, which bear grapes not only superior in flavour to others hitherto known, but which are capable of arriving at perfection, even in the most adverse seasons, in our climate. For these, and other discoveries, ably enumerated by the learned President, the Copleyan Medal was adjudged to Mr. Knight, whose successful labours in this branch of natural history have probably surpassed those of any other philosopher, in developing the economy of vegetation, and the laws of vegetable life.

M. L. ABBE MELOGRANI has invented a new Blow-pipe: it consists of two hollow glass globes, of a size proportioned to the effect required, which are united by two metallic tubes placed one against the other; each of these pipes has a valve attached at each of its extremities: a third pipe placed horizontally, and at right angles with the two first, is hermetically fixed to the pipes which unite the two globes. This horizontal pipe, besides serving to direct the air upon the flame of the lamp, likewise forms a support and axis on which the globes turn. When the lower globe, which is half filled with water, has, in changing its position, become uppermost, the water will run out into the other, and will form, by the pressure, a current of air in the pipe, which being stopped by the valve at the extremity of the same pipe, will be forced to pass through the horizontal pipe; the mouth of which being directed towards the flame, will produce the effect desired: when the water has descended into the lower ball, the position must be changed, and the action of the machine will recommence.

M. THEODORE PIERRE BERTIN has invented a new syphon, capable of raising water thirty feet high without human help. This instrument is, we are told, applicable to different purposes: As a syphon, it may be used to raise water above its source, in any situation; as a pump, it may serve as a pneumatic chemical apparatus, by the help of which may be made acidulated waters. The effects of this pump are in proportion to the superior length of the descending limb over that of the ascending one: it is therefore convenient for conveying perfumed air, such as that of an orangerie, for example, into rooms; it may also be rendered useful for mild suctions, and might be employed in surgical operations where the sucking pump is employed.

Dr. CARRADORI, in opposition to the experiments and conclusions of Messrs. Humbolt and Gay Lusac, affirms that ebullition is not sufficient to free water from all the oxygen that it contains; and that nothing but congelation, and the respiration of fishes, can entirely clear water of its oxygen. These, he says, are the only means that complete the separation from water of all the oxygen it contains interposed between its globules. Fishes he conceives to be the eudiometers of water; and one of these, shut up in a body of water, is capable of separating, by means of its respiration, in several hours, all the oxygen from the water, and to exhaust it entirely from this principle. By several ingenious but cruel experiments on fish, this philosopher proves that melted snow, as well as water that has been congealed, is deprived of all its oxygen.

Mr. HERMBSTADT, of Berlin, gives the following as a cheap Method of obtaining the Sugar of the Beet-root:—Let the beet-roots be pounded in a mortar, and then subjected to the press; the juice is next to be clarified with lime, like that of the sugar-cane, and then by evaporation bring it to the consistence of syrup. From 100lbs. of raw sugar thus obtained, 80lbs. may be had by the first refining, of well-crystalized sugar, inferior neither in quality nor whiteness to that of the West-Indies. Two days are sufficient to complete the operation.

Mr. DAVY, in the concluding Lecture of the first part of his Course on Vegetable Chemistry, gave a new theory to account for the Fairy Rings, so common in some meadow lands. They have of late years, been generally supposed to be occasioned by the electric fluid; but, according to Mr. Davy, every fungus exhausts the ground on which it grows, so that no other can exist on the same spot: it sheds its seed around, and on the second year, year, instead of a single fungus as a centre, a number arise in an exterior ring, around the spot where the individual stood: these exhaust the ground on which they have come to perfection; and, on the succeeding year, the ring becomes still larger, from the same principle of divergency. Mr. Davy acknowledged himself indebted to Dr. Wollaston for this ingenious theory.

Mr. Davy, in the concluding part of a Paper lately read before the Royal Society, considered the influence of electricity in the mineral kingdom; its action on the carbonet of iron, and various other mineral bodies; and also its importance, as tending to elucidate many phenomena in the science of geology. In the course of this Paper, were detailed an account of several original experiments on the effects of electricity on certain chemical menstrua; in all of which the negative pole disengaged oxygen, and the positive hydrogen.

It has been lately recommended that, excepting the lancet employed in vaccination, all the instruments of surgery ought to be dipped into oil at the moment when they are going to be used; by which method the pain of the subject operated upon will always be diminished. It is recommended to make all instruments of a blood-heat a little before the operation.

M. Seguin, from the remarkable quantity of albumen found in vegetable juices which ferment without yeast, and afford a vinous liquor, has been led to enquire whether the albumen might not be of essential consequence to this intestine motion. Having deprived these juices of albumen, they became incapable of fermenting; and then having supplied this principle, such as white of egg, to saccharine matter, the fermentation took place, and a matter similar to yeast was deposited, which appeared to be only the albumen, which was so altered as to be nearly insoluble, without having lost its fermentescible action. Hence he concludes, that albumen, whether animal or vegetable, is the true ferment.

The London Medical Society propose to confer the Fothergillian gold medal upon the authors of the best essays on the following subjects.

*Questions for the year* 1807.—The best account of the epidemic fevers which have prevailed at several times in North America, Spain, and Gibraltar, since the year 1793, and whether they are the same or different diseases?

*For the year* 1808.—What are the best methods of preventing and of curing epidemic dysentery?

*For the year* 1809.—What are the criteria by which epidemic disorders that are not infectious may be distinguished from those that are?

*For the year* 1810.—What are the qualities in the atmosphere most to be desired, under the various circumstances of pulmonary consumption?

Mr. Lansdown's Method of preparing Canvas, so as to make flexible Tubes for conveying fresh Air into Coal Mines, and for other other useful Purposes.—Take one quart of boiled linseed oil, (that is, I suppose linseed oil boiled with litharge, &c.) and a quarter of a pound of the gum elastic; boil them together; they will so boil near two hours, before the gum will be dissolved; then add three quarts more of boiled oil, one pound of resin, one pound of bees wax, and one pound of litharge of lead; boil all up together, and with a brush lay it warm and evenly, as a coat on the canvas. This prepared substance will be as flexible as can be wished, without cracking; will resist wet or damp, and will be found durable, if common prudence be exercised in the care of it.

The secret of the Invisible Girl has lately been supposed to have been discovered, from which it should seem, that the whole deception consists in a very trifling addition to the mechanism of the Speaking Bust; which consists of a tube from the mouth of the bust, leading to a confederate in an adjoining room, and another tube to the same place, ending in the ear of the figure. By the last of these a sound whispered to the ear of the bust, is immediately carried to the confederate, who instantly returns an answer by the other tube ending in tbe mouth of the figure, who seems to utter it; and the invisible girl only differs in this circumstance, that an artificial echo is produced by means of certain trumpets; and thus the sound does not proceed in its original direction, but is completely reversed.

---

*St. Thomas's and Guy's Hospitals.*

The following Lectures compose the Spring Courses at these Hospitals.

Anatomy, and the Operations of Surgery, by Mr. Cline and Mr. Cooper.

Principles and Practice of Surgery, Mr. Cooper.

Practice of Medicine, Dr. Babington and Dr. Curry.

Pathology, Therapeutics, and Materia Medica, Dr. Curry.

Chemistry, Dr. Babington and Mr. Allen.

Experimental Philosophy, Mr. Allen.

Physiology, or Laws of the Animal Economy, Dr. Haighton.

Midwifry, and Diseases of Women and Children, Dr. Haighton.

Odontology, or Structure and Diseases of the Teeth, Mr. Fox.

N. B. None of the Lectures given at these Hospitals, interfere with each other in the hours of attendance; and the whole is calculated to form a Complete Course of Medical and Surgical Instruction. Terms and other particulars to be learnt of Mr. Stocker, Apothecary to Guy's Hospital; who is also empowered to enter Gentlemen as Pupils to such of the Lectures as are delivered at Guy's.

Mr. Chevalier, Surgeon Extraordinary to the Prince of Wales, and Surgeon to the Westminster General Dispensary, will commence his next Course of Lectures on the Principles and Operations of Surgery, on Monday the 9th of February, at eight o'clock o'clock in the evening, at his house in South Audley-street, Grosvenor-square, where printed particulars may be had.

Dr. John Reid will commence a Course of Thirty-five Lectures on the Theory and Practice of Medicine, on Friday the 6th of February, at his house in Grenville Street, Brunswick Square.

Mr. Milburne's Lectures on the Principles and Operations of Surgery, will commence on Monday the 9th instant.

Mr. Moor, Surgeon Dentist to her Royal Highness the Duchess of York, will commence his Spring Course of Lectures on the Structure and Diseases of the Teeth, on the 14th of February, in which will be explained the Complete Practice of the Dentist; further particulars may be known, by applying at his house, No. 6, Palsgrave Place, Temple.

Mr. Lawrence, of the Anatomical Theatre, St. Bartholomew's Hospital, has in the press, a Translation from the German of Blumenbach's Comparative Anatomy, with numerous additional Notes.

Dr. Bardsley, Physician to the Manchester Infirmary, has committed to the press, and will speedily publish, a Selection of Medical Reports of Cases, Observations, and Experiments, chiefly derived from hospital practice; including, among others, Clinical Histories of Diabetes, Chronic Rheumatism, and Hydrophobia.

Dr. Kinglake is preparing some Strictures on Mr. Parkinson's Observations on the Nature and Cure of Gout, recently published in opposition to the theory that proposes the cooling treatment of that disease. The same gentleman is about to publish additional Cases of Gout, in farther proof of the salutary efficacy of the cooling treatment of that afflicting disease; with illustrative annotations, written authorities in its support, controversial discussions, and a view of the present state and future prospects of the practice. And also a pamphlet, called the Reviewers Reviewed, containing general observations on legitimate and licentious criticism.

Dr. A. P. Wilson of Worcester, has nearly ready for publication an Essay on the Nature of Fever.

---

